# Tumour progression and metastasis

**DOI:** 10.3332/ecancer.2016.617

**Published:** 2016-01-29

**Authors:** Francisco Arvelo, Felipe Sojo, Carlos Cotte

**Affiliations:** 1 Centro de Biociencias, Fundación Instituto de Estudios Avanzado [IDEA], Caracas 1015-A, Venezuela, Apartado 17606, Caracas 1015-A, Venezuela; 2 Laboratorio de Cultivo de Tejidos y Biología de Tumores, Instituto de Biología Experimental, Universidad Central de Venezuela, Apartado 47114, Caracas, 1041-A, Venezuela

**Keywords:** cancer, infiltration, metastasis, microenvironment, metastatic niche, latency, epithelial–mesenchymal transition

## Abstract

The two biological mechanisms that determine types of malignancy are infiltration and metastasis, for which tumour microenvironment plays a key role in developing and establishing the morphology, growth and invasiveness of a malignancy. The microenvironment is formed by complex tissue containing the extracellular matrix, tumour and non-tumour cells, a signalling network of cytokines, chemokines, growth factors, and proteases that control autocrine and paracrine communication among individual cells, facilitating tumour progression. During the development of the primary tumour, the tumour stroma and continuous genetic changes within the cells makes it possible for them to migrate, having to count on a pre-metastatic niche receptor that allows the tumour’s survival and distant growth. These niches are induced by factors produced by the primary tumour; if it is eradicated, the active niches become responsible for activating the latent disseminated cells. Due to the importance of these mechanisms, the strategies that develop tumour cells during tumour progression and the way in which the microenvironment influences the formation of metastasis are reviewed. It also suggests that the metastatic niche can be an ideal target for new treatments that make controlling metastasis possible.

## Introduction

**Figure 1.**
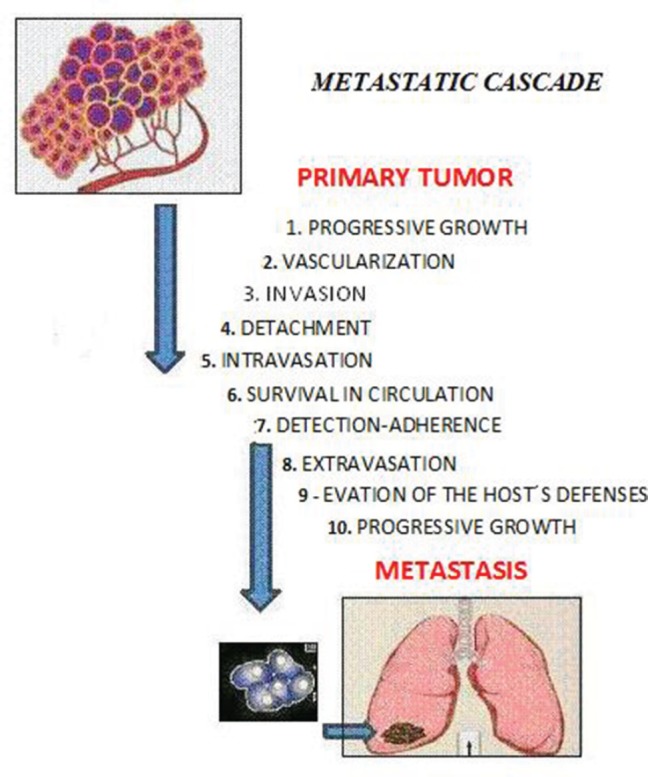
The sequential steps in the pathogenesis of metastasis are shown in the figure above, where each step is regulated by transitory and permanent changes in the DNA, the RNA, or by proteins. Also, most tumor cells fail to complete all of the steps, and the ‘few’ cells with metastatic ability ‘defeat’ the multiple mechanisms which impedes the formation of metastasis.

Metastasis is defined as ‘the process of dissemination of cancer cells from their origin to a distant organ’ [1], a complex process involving several stages, which are as follows: (a) the activation of epithelial–mesenchymal transition (EMT), during which cancer cells lose all cell–cell contact, such as substrate adhesion, acquiring ownership of movement; (b) local invasion, whereby malignant cells degrade the basal lamina, the special extracellular matrix that organises and separates epithelial tissues from the stroma, which plays an important role in both cell signalling and being a reservoir of growth factors released by tumour cells; (c) intravasation, during which tumour cells pass through the walls of blood vessels and enter the bloodstream; (d) the ability to survive in the bloodstream; (e) extravasation, whereby tumour cells exit the bloodstream, passing through the walls of blood vessels into the tissue of a particular organ; (f) establishment of tumour cells in the tissues of the organ where metastasis will form; in other words, the establishment of a pre-metastatic niche to create a favourable environment for the growth of cancer cells (Figure 1).

Each of the steps necessary in producing metastasis, from the arrival of malignant cells to their growth and proliferation in the host organ, is led by the genetic and/or epigenetic alterations acquired and accumulated during the course of tumour progression [2, 3]. Despite recent advances made in surgical techniques, radiotherapy and the development of molecularly targeted therapies, the majority of cancer-related deaths (more than 90%) are the result of progressive growth of therapy-resistant metastasis [4]. Metastatic cells come from a heterogeneous cell population within the primary tumour which, over time, are selected, experience a high spontaneous mutation rate, are more prone to suffering from rapid phenotypic diversification and are resistant to therapeutic treatments [5].

The survival of the malignant cells that form micrometastasis in the receptor organ is not ensured, as there may be differences between the primary tumour’s microenvironment and the location where the cancer cells will be established [6]. Therefore, the pre-metastatic niche model has been proposed, which can be described as ‘the location with the necessary microenvironmental conditions for the disseminated tumour cells’ survival’ [7]. For adaption to occur, they deploy mechanisms to modify the new microenvironment. To do so, along with the stroma cells, they establish a signalling network to promote their growth, to satisfy the metabolic demands to synthesise proangiogenic proteins in order to form new vascular networks and to facilitate their initial survival in the new ectopic location [8].

Metastasis is a largely ineffective process, leading it to be called ‘metastatic inefficiency’, since only 0.01% of the tumour cells that enter the bloodstream in animal models successfully form a secondary tumour [9]. With these models, it has been possible to choose cell populations with metastatic phenotypes and these cells in particular show increasing selectivity after each selection cycle for a specific organ [10]. Furthermore, the genome instability of neoplasms increases the likelihood of other cells developing skills that allow for the development of metastasis. Several studies have shown that an increase in metastatic ability is not the result of the adaption mechanisms of the tumour cells that allows for growth in a specific organ; rather, it is the gradual selection of a clone with different mutations to those observed in the primary tumour. The genome instability and heterogeneity of cancer cells is shown in the gains, losses, and chromosomal rearrangement of the tumours [11]. New technologies in parallel sequencing have allowed high-resolution genome analysis of cancer patients, making it possible to compare the primary tumour with regard to metastasis.

These studies have proven that there are inactive genes in the primary tumour, while they are active in metastasis, allowing for the activation of other oncogenes, confirming the concept of tumour heterogeneity [12]. Additionally, it has been shown that mutation patterns are shared in the metastasis of an organ, whereas metastasis in the different organs of a patient is different, establishing the hypothesis that metastasis results from clonal expansion where the subclones that have colonised an organ already have genetic alterations that allow specific adaption to the environment [13]. The period of time that passes between infiltrating the organ and colonising it is known as the latency period, where several tumour cells remain outside the cell cycle of secondary organs, while others are incapable of provoking the angiogenic changes necessary for tumour expansion [14].

**Figure 2.**
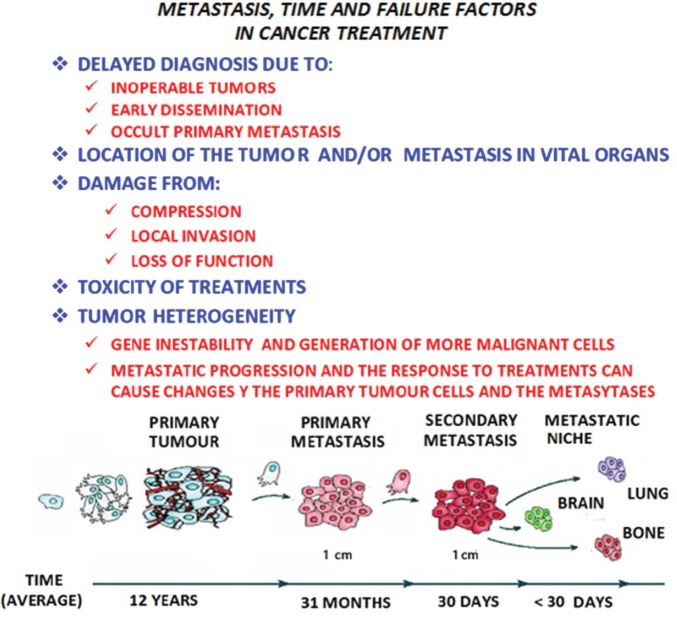
In metastatic progression, time and the failure factors indicated in the figure above, play a crucial role in the prognosis and treatment of the patient with cancer, as shown in the model of the linear progression of a tumor. In time, the cells increase in malignancy. Thus the concept of ‘metastasis of metastasis’ is used in reference to the metastasis becoming in the course of time more malignant, resulting in the death of the patient, as the illness cannot be controlled.

It would appear that metastasis is a process determined by a complex network of interactions between metastatic cells and their microenvironment in the affected organs, making it necessary to update the contributions made for the knowledge of the different microenvironment elements involved in the formation of metastasis. It is necessary to focus on the main interactions established between the tumour cells and the microenvironment, from the primary tumour to reach the location where metastasis originates and develops. In all these phenomena, time has to also be considered as a factor of singular and paramount importance (Figure 2).

## Components of the pre-metastastic niche

The metastatic niche plays an important role within the crucial factors that determine the success or failure of metastasis. A series of events take place in its formation, including: the modification of the extracellular matrix [ECM]; the restructuring of the vascular network; the participation of bone marrow cells; hypoxia and the expression of a wide variety of signalling molecules. Added to this is the participation of untransformed cells, such as fibroblasts and endothelial cells, as well as the deposition of molecules such as fibronectin, tenascin-c, and periostin [15]. Regarding particular tissue receptors, fibulin-5 reduces its levels so that matrix metallopeptidase 9 [MMP-9] restructures the matrix in metastasis in the liver and lungs, thus contributing to the formation of the metastatic niche [16]. Lysyl oxidase [LOX] actively participates in restructuring the extracellular matrix and the formation of the niche, so it has the ability to be linked to collagen and elastin. The expression of LOX is increased in tumour cells exposed to hypoxia conditions [17]. The development and consolidation of these investigations originated from the pioneering work made by Kaplan and co. [18], who used as many human tissue samples from cancer patients as Lewis lung carcinoma (LLC) and B16 cell lines from an animal model. They show that haematopoietic bone marrow-derived progenitor cells, which express vascular endothelial growth factor receptor 1 (VEGFR-1), form aggregated cells in pre-metastatic niches. The use of specific antibodies against VEGFR-1, as well as the elimination of these aggregated pre-metastatic cells, impedes the formation of metastasis. They also studied the adhesion and formation of aggregated cells after the implantation of the tumour cells, observing a growth in the expression of fibronectin and an increase in the dissemination of fibroblasts found in the primary tumour.

Furthermore, matrix metallopeptidases – particularly MMP-9 produced by bone marrow-derived progenitor cells – degrade the basal membrane, accelerating the extravasation of cells with VEGFR-1^+^ phenotype in the niche. Along with fibronectin, associated with stroma cells, VEGFR-1^+^ alters the local environment, activating integrin and chymosin to promote adhesion, survival, and growth in tumour cells. The primary tumour induces the production of MMP-9 in endothelial cells and macrophages in the lungs via a mechanism dependant on VEGFR-1/FLT-1 tyrosine kinase (TK) that promote metastasis. Additionally, they studied the expression of MMP-9 in healthy areas of the lungs in samples extracted from patients with tumours located in organs besides the lungs. In the lungs of patients with oesophageal cancer, melanoma skin cancer, ovarian cancer etcetera, a high expression of MMP-9 was found which suggests that primary tumours can stimulate the production of MMP-9 in pre-metastatic areas [19].

## Types of cells

There are several types of cells that are important components of the metastatic niche, including heterogeneous populations in immune cells, endothelial cells, fibroblasts, macrophages, pericytes, and bone marrow-derived progenitor cells, which are described below:

### Immune cells

They are represented by cells that actively participate in immunity processes, including macrophages, neutrophils, mast cells, myeloid-derived suppressor cells, dendritic cells, natural killer cells (NK) and more adaptive immune cells, such as T- and B-type lymphocytes. Immune cells that infiltrate the tumour, excluding NK cells, produce tumour cytokine promoters, such as tumour necrosis factor alpha [TNF-α] and interleukins IL-1β, IL-6, and IL-8, which increase signalling in pre-malignant cells. Likewise, this signalling not only stimulates tumour progression but also induces the production of cytokines by the tumour cells themselves [20].

### Neutrophils

They are the most abundant human leukocytes as they are the first cells that gather at the infection site. Their degranulation releases lytic enzymes, as well as reactive oxygen species (ROS), hydrogen peroxide (H_2_O_2_), and hypochlorite (HOCL) with microbial potential [21]. Cytokines are also produced, such as TNF-α, IL-1β, IL-1Rα, IL-12, and VEGF; chemokines CXCL1, CXCL8, CXCL9, CXCL10, CCL3, and

CCL4 are involved in angiogenesis [22]. CXCL8 is produced in abundance by tumour cells, which once released into the microenvironment represent a potent chemo-attractant of neutrophils within the tumour. Furthermore, CXCL8 and other chemokines are associated with angiogenesis by the direct extracellular activation of CXCR2 [23], in particular, MMP-9 [24]. TNF-α, released in the tumour microenvironment, is linked with tumour progression inducing degranulation of the neutrophils, releasing VEGF and favouring angiogenesis with the production of CXCL8 and CXCL1 [25].

### Dendritic cells

They represent a heterogeneous population made up of two types of cells: myeloids CD11c^+^ and CD123^lo^, and plasmacytoids CD11c^−^ and CD123^hi^ [26]. It has been shown that in various types of tumours, they present specific modifications in its stimulating ability with the consequent abnormal development of the differentiation of myeloid cells [27]. One of the mechanisms by which there is an abnormal differentiation of myeloid cells is the constitutive activation of the signal transducer and activator of transcription 3 [STAT3], which promotes continuing proliferation and the accumulation of immature myeloid cells, contributing to the suppression of the immune response before tumour angiogenesis [28]. Two pro-inflammatory molecules released by dendritic cells, TNF-α and osteopontin for instance, are associated with angiogenesis [29, 30]. Dendritic cells can secrete pro-angiogenic chemokines, such as CXCL1, CXCL2, CXCL3, CXCL5, CXCL8, and CCL2 [31]. In contrast, mature dendritic cells can inhibit angiogenesis by releasing cytokine IL-12 and chemokines CXCL9, CXCL10, and CCL21 [32].

### Myeloid-derived suppressor cells (MDSC)

They represent an active role in the promotion of tumours and the evasion of the immune response. When they are immature cells, they seem to have monocyte/macrophage and granulocyte characteristics [33]. High levels of pro-inflammatory factors in the microenvironment of the tumour, such as GM-CSF, IL-1b, IL-6, and S-100, induce the recruitment and expansion of myeloid-derived suppressor cells, increasing pro-tumour activity [34]. Myeloid-derived suppressor cells also participate in the promotion of angiogenesis in the tumour through the release of soluble factors like MMP9 and VEGF. Experimental data suggest that these cells are also capable of being different from endothelial cells [35].

### Natural killer cells

Natural killer (NK) cells are lymphocyte effectors of the innate immunity that could potentially control tumours by their cytotoxic activity. Like other types of cells, NK cells can infiltrate the mass, whose microenvironment is capable of affecting the functionality of these cells by a broad range of cytokines and soluble factors, either by inhibiting the cytotoxic function or promoting angiogenic phenotype. CD56^bright^ and CD16^-^ NK cells predominant in lung cancer exert lower cytotoxicity in K562 cells [36]. It has been reported that the invasion of NK cells in lung cancer (non-small-cell lung cancer [NSCLC]) produces high levels of VEGF, PIGF, and IL-8, inducing ‘*ex vivo’* angiogenic activity [37]. The reduction in activity by NK cells is associated with the generation of the pre-metastatic niche and the efficiency of metastasis in murine models [38].

### T cells

The inhibition of the flow of T-lymphocytes during angiogenesis and stroma restructuring represents a characteristic of the tumour microenvironment, giving way to alterations to its functionality. This is due to the activation and expansion of myeloid cells and soluble factors secreted by the tumour and inflammatory cells. The typical immunosuppressive tumour environment is characterised by a strong induction by CD4+, CD25+, FOXP3, and tumour-infiltrating regulatory T cells, and the activation of Th2 and Th17 [39, 40]. In ovarian cancer, hypoxia induces angiogenesis in humans and mice, where CD4^+^, CD25^+^ and tumour-infiltrating regulatory T cells secrete high quantities of VEGFA and promote the dissemination of endothelial cells, both ‘*in vitro*’ and ‘*in vivo*’. In an ovarian tumour transplanted in a mouse, the depletion of tumour-infiltrating regulatory T cells correlates with a reduction in VEGFA, supporting the role of tumour-infiltrating regulatory T cells in angiogenesis in this type of tumour [41].

### B cells

The stimulation of B cells culminates in the production of immunoglobulins [I_g_] that participate in humoural immunity. They segregate a variety of cytokines such as interleukins IL-6 and IL-10, TNF-α, granulocyte-macrophage colony-stimulating factor (GM-CSF), and lymphotoxin (LT) that participate in humoural immunity [42]. In the development of solid tumours with similar characteristics to damaged tissues with immune dysfunction, such as chronic immune cell infiltration, tissue restructuring or angiogenesis, it is unsurprising that individuals with autoimmune diseases are at increased risk of developing cancer [43]. Complement proteins are associated with immunoglobulins and form circulating immune complexes (CIC), whose deposition in the parenchyma is due to defects in the vascular network, either by the tumour or by pathological angiogenesis, which starts a chain reaction [44]. In certain types of cancer, CIC levels in the tumour parenchyma are linked to an increase in tumour burden, indicating a poor prognosis [45]. In the K14-HPV16 mouse model, a carrier of squamous cell carcinoma (SCC), the suppression of B- and T-lymphocytes produced a decrease in angiogenesis and epithelial hyperproliferation. The transfer of B cells (B220^+^ and CD19^+^) in K14-HPV16 mice to K14-HPV16 mice (sic), deficient in T- and B-lymphocytes, restored malignant characteristics, such as hyperproliferation and angiogenesis. These data indicate that the activation of B cells is essential for the development of an epithelial neoplasm and that soluble mediators secreted by B cells are necessary to establish an inflammatory process that boosts tumour progression [46].

### Mast cells

They represent a peculiar subtype of granulocytes that play a central role in the inflammatory process, participating in vascularisation during arthritis [47]. They have also been found to participate in the vascularisation of haematological malignancies, where they can integrate in the blood vessel wall by vascular mimicry [48]. The participation of mast cells in angiogenesis is associated with the production of various cytokines and chemokines [49]. Furthermore, proteases produced by mast cells promote angiogenesis [50]. B-triptase is a neutral protease that represents an abundant mediator found next to mast cell granules and plays an important role in inflammation, activating the release of protease by type 2 receptors that are directly involved in vascularisation [51].

## Tumour-associated macrophages (TAM)

They act as regulators of tumourigenesis, either as residents or as derived from the spleen or bone marrow. Macrophages are typically considered as effector cells during immune defence but numerous studies have shown their role in tumour progression [52]. They are an important source of proteases, such as cysteine and cathepsin, that participate in tumour progression [53]. Tumour-associated macrophages have antagonistic functions between the homeostasis of normal tissues and tumorigenesis, which is why macrophages are functionally plastic and can alter their phenotype to suit different physiological conditions [54]. They can present a ‘M1’ phenotype that produces a type 1 pro-inflammatory cytokine that participates in antigen presentation, playing an antitumorigenic role, as well as a ‘M2’ phenotype that produces a type 2 cytokine that promotes the anti-inflammatory response and pro-tumorigenic function [55]. It has been suggested that in several environmental conditions, such as tumour hypoxia in a ‘M1’ to ‘M2’ transition, tumour-associated macrophages accumulate in hypoxic areas within the tumour, as well as endotheline-2 and VEGF. It should be noted that the accumulation of tumour-associated macrophages is linked with angiogenesis and the subsequent acquisition of invasive phenotypes [56]. Furthermore, a population of metastasis-associated macrophages (MAM) has been identified in a mouse model that promotes extravasation, dissemination and growth of breast cancer cells in the lungs. The inhibition of signalling by CCL2–CCR2 inhibits the accumulation of metastasis-associated macrophages and reduces metastasis [57]. Blood clotting plays an important role during metastasis, due to the blood-clotting proteins and tissue factor (TF) being linked to a poor prognosis for patients as they interfere with NK cells through the lysis of micrometastases. TF induces the formation of blood clots with platelets that stimulate the recruitment of bone marrow-derived macrophages, resulting in the survival of melanoma cells in the lungs. These blood clots recruit myeloid-derived suppressor cells in secondary niches, impeding the immunological rejection of the tumour [58].

## Cancer-associated fibroblasts (CAF)

They are predominant cells in the connective tissue responsible for the elaboration of the extracellular matrix components and basal membrane, associated with the differentiation of epithelial cells and mediators of the immune response [59]. There are numerous cancer-associated fibroblasts in the tumour microenvironment, differing from normal fibroblasts. In mice, normal prostrate epithelial cells originate in intraepithelial tumours when coinjected with cancer-associated fibroblasts, but not when they are injected with normal fibroblasts [60]. Likewise, in breast cancer, cancer-associated fibroblasts stimulate the metastasis of malignant cells, while normal fibroblasts suppress metastasis [61]. This shows that cancer-associated fibroblasts make up a cell different to its normal counterpart. Moreover, their origin during the progression of the disease is unclear [62]; several studies suggest that they generate through the epithelial–mesenchymal transition of endothelial cells from blood vessels associated with tumours [63]. The mesenchymal–epithelial transition (MET) promotes cancer-associated fibroblast generation in tumours of epithelial origins; for instance, in breast cancer and prostate cancer, epithelial cells dedifferentiate to generate mesenchymal cells that express cancer-associated fibroblast markers [64]. Cancer-associated fibroblasts interact with tumour cells and additional components of the stroma through the production and secretion of various growth factors, cytokines, and chemokines. Once activated by the infiltration of immune cells, these fibroblasts produce pro-inflammatory chemokines such as CXCL1 and CXCL2 through the recruitment of tumour-associated macrophage*s* in primary tumours [65], while CCL5 being secreted by these fibroblasts recruits tumour-infiltrating regulatory T cells by signalling through the CCR1 receptor expressed in these cells [66]. CCL5 is secreted by mesenchymal stem cells (MSC) that also act through the CCR5 receptor expressed by breast cancer cells, increasing the invasion and metastasis [67]. Moreover, CXCL12 and fibroblast growth factor receptor 2 (FGF-2), released by cancer-associated fibroblasts, stimulate neoangiogenesis by recruiting endothelial progenitor cells and vascular endothelial cells [68]. In mesenchymal–epithelial transition, tumour-associated fibroblasts are activated by TGF-β, PDGF, FGF, and proteases [69]. Once activated, cancer-associated fibroblasts secrete growth factors, including VEGF that induces vascular permeability and angiogenesis [70, 71].

## Pericytes

They are specialised mesenchymal cells that are linked to smooth muscle, which act as support to endothelial cells and contribute both towards homoeostasis and the stabilisation, maturation and restructuring of capilliaries [72]. The intimate anatomical relationship between endothelial cells and pericytes suggests a stretched interaction between cell contacts by paracrine signalling. Platelet-derived growth factor B (PDGFB) is a family member of PDGF secreted by endothelial cells that joins with the tyrosine kinase receptor, PDGFR, expressed on the surface of pericytes. When PDGFB joins with PDGFR, dimerisation occurs and an intracellular signalling cascade that promotes cell proliferation and migration begins [73]. Angiopoietin-1 (Ang-1) is a soluble ligand produced by pericytes that joins with the tyrosine kinase receptor Tie-2, expressed by endothelial cells [74]. The interaction between Ang-1 and Tie-2 is fundamental for the maturation and stabilisation of the endothelium [75]. Transforming growth factor β (TGF-β) is a growth factor expressed by endothelial cells and pericytes during angiogenesis [76]. Vascularisation in tumours is chaotic and irregular, an instability that has been frequently attributed to a reduction in the number of pericytes [77]. The presence of pericytes can vary according to the type of tumour, considering that they increase in pancreatic cancer for example and decrease in glioblastoma, a notable fact when compared with the respective normal tissues. In reality, they are found in the majority of tumours, even though their association with the endothelium is abnormal [78]. Different studies have shown that they are essential in maintaining the tumour vascular network, as well as normal blood vessels, while the VEGF produced by the pericyte is necessary for the survival of endothelial cells in both contexts [79]. A hypothesis considers that the reduction of the number of pericytes in tumour vessels can increase intravasation of cancer cells, promoting its haematogenous dissemination [78]. In fact, it has shown the existence of an inverted link between the contents in pericytes of tumour vessels and the number of metastasis in colorectal cancer patients [80].

## The composition of an inflammatory environment

An inflammatory environment may be created with the establishment of a tumour [81], as it has been observed that in many cases a pro-inflammatory environment is created which is composed of cytokines, chemokines, growth factors, activated stroma, metalloproteinases which degrade the extracellular matrix, and agents that cause damage to the DNA [82]. The inflammatory microenvironment introduced by the tumour cells affects the immune effector functions by means of immunosuppressor cells of the tumour-associated macrophage (TAM) type, immature Grl^+^ and Mac1^+^ myeloid cells, T regulator lymphocytes, T reg, CD4^+^ CD25^+^, natural killer T, NKT, etc. It is also possible that this occurs through the reduction of the number of dendritic cells, which are essential to initiating and maintaining an antitumour immune response [83]. The acute phase C-reactive protein or CRP and the A-amyloid or SAA proteins play a very important role in the induction of this inflammatory medium [84]. The SAA proteins are also chemotactic for other inflammatory cells such as the mastocytes and T lymphocytes [85], as well as in the induction of the expression of enzymes for the remodelling of the extracellular matrix [86] and in the production of inflammatory cytokines, such as TNF-α, that promote tumour growth [87]. The CD11b^+^ Gr1^+^ myeloid suppressor cells inhibit the T and NK cells, protecting the tumour cells from immunological destruction [88]. The T regulator cells (T_reg_) are found in the tumour microenvironment and have several immunomodulator activities in cancer [89]. In normal physiological conditions, the T_reg_ cells regulate the expansion and activation of the T and B lymphocytes, playing a critical role in the cytotoxic homoeostasis of the lymphocytes [90]. Based on the response to different environmental stimuli, the T_reg_ cells have different effects on tumorigenesis, for example in breast tumours, which show that an increase in T_reg_ cells is correlated with a lower survival [91], while in colorectal cancer, the T_reg_ cells are associated with a higher survival [92]. Similar to the myeloid-derived suppressor cells or MDSCs, the T_reg_ cells suppress the introduction of tumour-associated antigen and also interfere with the function of the cytotoxic T cells by means of inhibition through the release of cytolytic granules [93]. The S100A8 and S100A9 proteins, produced by primary tumours, induce the accumulation of haematopoietic progenitor cells and macrophages in pre-metastatic regions of the lung. In these regions, the serum amyloid A (SAA)3, induced by S100A8 and S100A9, acts as a regulating agent in the accumulation of the myeloid cells. In the lung, in the endothelial cells and macrophages, the toll-like4 (TLR4) receptor acts as a receptor for A(SAA)3 in the pre-metastatic phase, which also stimulates the signalling of NFKappa B, facilitating metastasis. This pro-inflammatory condition accelerates the migration of the primary tumour cells to the pre-metastatic niche in the lung [94]. In an animal model with metastasis in the lung, significant changes were observed in the vascular permeability that contributes to the establishment of the metastasis. The MD2 receptor represents a TLR4 coreceptor that creates regions of hyperpermeability by means of the overregulation of the CCR2 chemokine receptor. The CCR2-CCL2 system induces the secretion of permeability factors such as serum amyloid A3 and S100A8. This result suggests the possibility that the overregulation of CCR2 represents a marker for the regions of greater susceptibility to metastasis in lung cancer [95].

## The epithelial–mesenchymal transition

Local invasion implies profound changes in the adhesion and the proteolytic and migratory properties of the tumour cells, which favours cellular disassociation, degradation of the extracellular matrix, and migration to adjacent tissues. In addition, the excessive proliferation of epithelial cells and angiogenesis are the markers of initiation and growth, which can be observed in a primary carcinoma [96]. During the progression of a carcinoma, the already differentiated tumour cells alter their genome, which confers an advantage on the cell in terms of growth. In later stages, the cells continue to change their genome and exhibit a non-differentiated phenotype frequently accompanied by a low expression of epithelial markers, which leads to a loss of intercellular junctions and epithelial polarity. These changes are often accompanied by an increase in the expression of mesenchymal markers, as well as the mobility of the cells, which gives them greater invasive capacity. The process by which the cells change from an epithelial phenotype to a mesenchymal phenotype is known as the epithelial–mesenchymal transition or EMT. This process can be defined as a cellular programme that permits the phenotype transition of the cell from epithelial to mesenchymal [97, 98].

The marginal zone of the tumour is an active and interactive region of great importance to the tumour microenvironment, where immune cells and stromal cells accumulate. In the case of the immature myeloid cells that accumulate in this region, they impede the differentiation of the antigen initiator cells, thus favouring the evasion of the tumour cells [99]. The macrophages are another type of principal cells that are found in the marginal zone and are recruited by the products secreted by the tumour cells [100]. Studies have demonstrated the importance of the stroma during the epithelial–mesenchymal transition in cancer by means of the presence or absence of TGF-β [101]. In teratocarcinomas, the accumulation of macrophages induces the epithelial–mesenchymal transition due to the TGF-β factor produced by the macrophages associated with the tumours [102].

The epithelial–mesenchymal transition can also be induced by TGF-β secreted by the platelets [103]. The macrophages also promote the invasion of tumour cells by means of the supply of migratory factors, such as EGF, which through the regulation of the production of fibrillar collagen, accelerate cellular motility and induces proteolytic activity for the remodelling of the extracellular matrix [104]. During the EMT transition, alterations occur in cell–cell adhesion, cell–substrate interaction, degradation of the extracellular matrix, and the reorganisation of the cytoskeleton [105]. EMT is fully achieved when degradation of the basal membrane occurs and a mesenchymatic cell can migrate, this acquiring invasive capacity, which permits metastatic dissemination. The activation of the EMT programme has been proposed as a critical mechanism for the acquisition of the malignant phenotype by the epithelial cells [106].

The cells that initiate the EMT transition are found on the invasive front of the primary tumours expressing mesenchymal markers that are capable of effecting intravasation, as they are transported through the circulation, into micro or macrometastatic forms [6]. In addition, the metastasis and the primary tumours are histologically similar, which can be interpreted as a reversible EMT that would permit migration and dissemination to different organs in the first place. Once the cells that have undergone EMT are in place, they would activate the opposite programme, the MET transition, which would permit them to establish secondary colonies by returning to epithelial morphology and re-acquiring the ability to grow and proliferate [107].

## Transcriptional regulation of the epithelial–mesenchymal transition

A large number of molecular processes cooperate in the initiation and completion of the EMT transition, including the activation of transcription factors, the expression of specific surface proteins, the reorganisation and expression of proteins in the cytoskeleton, the production of enzymes that degrade the extracellular matrix, and the changes in the expression of miRNAs [108]. A key step in the EMT is the reduction in cell–cell adhesion by means of the transcriptional repression of the cadherins, components of the aherens junctions, such as occludin and claudin, components of tight junctions, to which is added the desmoplaquins, components of the desmosomes [109]. β-Catenin forms part of the adherens junctions, such that on breaking it migrates to the nucleus, where it functions as a cofactor for the T_cf_ or T-cell factor/lef transcription factors [110]. These then activate the transcription of genes such as c-myc, which increases cellular proliferation [111].

The expression of the intermediate filaments changes during the EMT with the substitution of keratin by vimentin [112], while the metalloproteinases increase during the EMT, participating in the loss of cell–cell junctions and the degradation of the basal membrane [113]. Several transcription factors that regulate the EMT inhibit apoptosis through activation of the MAPK and PI3K pathways [114]. During tumour progression, E-cadherin can be inactivated through the repression by means of the hypermethylation and deacetylation of the promoter through joining with the transcription receptors [115]. E-boxes, or short sequences of six nucleotides, -CACCTG or CAGGTG-, which determine specific expression in epithelial cells, were identified through analysis of the proximal promotor of E-cadherin in rats. The inactivation of these sequences activates the transcription of the E-cadherin in mesenchymal cells, which indicates the presence of repressors that silence the expression of this protein in non-epithelial cells [116]. The first repressors identified were the transcription factors with zinc fingers, Snail 1, and Snail 2 (slug) domains [117, 118], and the Zeb1 and Zeb2 transcription factors [119, 120], all of which are able to join with the E boxes of the cadherin promoter. Other repressors include the basic helix-loop-helix E12/E47 (TCF3) and Twist transcription factors [121, 122]. These repressors repress E-cadherin by means of the recruitment of some corepressors, such as for example, in the case of twist, activating the expression of other E-cadherin repressors. Furthermore, the overexpression of these factors in the epithelial cells not only produces the repression of E-cadherin, but also the reprogramming of the cell to a mesenchymal state. During the EMT, these repressors also repress other molecules from the adherens junctions and induce mesenchymal characteristics in a coordinated manner [123]. Also, the expression of Snail 1 induces the expression of fibronectin or vitronectin [124]. In the case of Twist, this regulator of the EMT induces the expression of the Akt kinase, a P13K effector and an important regulator in the survival pathways during the EMT [125].

The small non-coding RNa or microRNAs also act as regulators of the EMT transition, inhibiting the gene expression at the post-transcriptional level with the consequent reduction of the stability of the mRNAs, which are their target [126]. The constituents of the metastatic niche, as well as the remodelling of the ECM, have been associated with the induction of the EMT. Periostin or osteoblast-specific factor OSF-2 promotes its induction [127]. The EMT is induced by the metalloproteinases, MMPs that are activated in the metastatice niche [128]. Hypoxia also induces the activation of the EMT transition [129]. Other studies have demonstrated that the induction of EMT in the MCF10A cells with a high expression of SNAIL contributes to (a) resistance to antitumour drugs, (b) the acquisition of the mother cell phenotype through an increase in the expression of the CD44^+^/CD24^−^ surface markers, (c) the capacity to form mammospheres [130]. In the case of the resistance to drugs developed by the mother cells, this represents one of the greatest challenges for chemotherapy against cancer [131]. This mainly affects tumour cells with rapid proliferation, while the mother cells grow slowly and have an effective resistance mechanism. This resistance to treatment leads to an increase in the rate of proliferation of the mother cells, which results in a recurrence of the cancer and metastasis [132].

## Signalling pathways that activate the epithelial transition

The interaction of the tumour cells with the local microenvironment induces the autocrine or paracrine secretion of the growth factors, cytokines, and components of the extracellular matrix that can trigger the molecular programme for the EMT transition [133]. A large number of signalling pathways and growth factors have been associated with the EMT transition, such as the epidermal growth factor EGF, the fibroblastic growth factor FGF [134], and the hepatic growth factor or HGF [135]. The Wnt/β-catenin pathway is related to the EMT transition [136], while the TGF-β factor is another EMT inducer, as the signals activated by this factor inhibit some epithelial proteins, such as E-cadherin and keratin. They also activate the expression of mesenchymal proteins, such as fibronectin and vimentin, and in the case of TGF-β, it acts in the activation of the EMT through the Smad proteins [137]. Another pathway implicated in the induction of the EMT is that activated by NOTCH, which in turn is induced by hypoxia [138]. The NF-kβ factor acts as a regulator of the EMT, which is important for protection against apoptosis and in the induction of metastasis [139].

## The metalloproteinases

The metalloproteinases or MMPs belong to a family of zinc-dependent endopeptidases that intervene both in the physiological processes of organogenesis and in the scar formation and in various pathological conditions, particularly cancer [140]. Although the first well-studied function of the metalloproteinases is the degradation of the ECM, it is currently believed that they play an important role in the processing of bioactive molecules such as growth factors, cytokines and chemokines, as well as their respective receptors [141]. The MMPs modulate the mediators of inflammation, such as the cytokines and chemokines, establishing the necessary gradients for chemotaxis of the inflammatory cells. They facilitate the migration of epithelial cells by interacting with these and the ECM proteins through proteolysis of the same matrix or the tissue adhesion proteins, such as E-cadherins [142]. In the vascular system, they influence the migration of tumour cells, the liberation of cytokines and growth factors linked to the cellular membrane, such as the transforming growth factor alpha TGF-α and the epidermal growth factor EGF [143]. In many malignant tumours, there is overexpression of ADAMs, desintegrin, and metalloproteinase, where their role in tumour growth and dissemination is related to their proteolytic activity [144]. Several MMPs and MT-MMPs have an important activating action on other pro-MMPs, while they are activated by other proteases. These processes usually take place in the extracellular space, but there is a group of proteases, the MT-MMPs, MMP-11, MMP-23, and MMP-28, which activate within the cell by means of a furin-type proprotein convertase [145]. The MMPs also act as signalling molecules and can then modulate other cell signalling molecules. The platelet-derived growth factor PDGF produces an increase in the expression of MMP-1, which acts in conjunction with TGF-β, producing an overexpression of MMP-3 and TIMP-1 [146]. The epidermal growth factor EGF induces the expression of MMP-1 [147], and the vascular endothelial growth factor VEGF and fibroblast growth factor FGF-2 are angiogenic factors that can induce the expression of the MMP proteases, facilitating metastatic dissemination [148]. The tumour necrosis factor alpha TGF-α is a pro-inflammatory cytokine liberated by macrophages, T lypmhocytes, and mastocytes that induce the overexpression of some MMPs such as MMP-2, MMP-3, MMP-7, and MMP-9 in the tumour microenvironment, which increases the invasive capacity of the malignant cells [149].

## The metalloproteinases and the progression of cancer

The MMPs are the principal mediators in the changes observed in the tumour microenvironment during the progression of cancer [150], which play a fundamental role in tumour growth through degradation of the connective tissue and the components of the basal membrane, in addition to activating growth factors, surface receptors for adhesion molecules and for chemokines [151]. This interaction with the components of the ECM alters the cellular response to the microenvironment, making the tumour cells less adherent so they have more potential to migrate and produce metastasis [152]. During carcinogenesis, the tumour cells interact with the growth factors, the cytokines, and other cells, such as endothelial cells, fibroblasts, macrophages, mastocytes, and pericites, that are present in the tumour microenvironment [153]. All of this demonstrates that the remodelling of the ECM is an active event during tumour progression that leads to the formation of a niche for the survival and proliferation of the tumour cells [154].

The MMPs can play different roles during the progression of the cancer, depending on the stage of the tumour. In early stages, the proteolysis of MMPs 3 and 7, which link growth factors, contribute to cellular proliferation, but later, the cleavage of E-cadherin and CD44 activates the motility of the tumour cells facilitating metastasis [155]. In contrast, MMP-8 has a protective effect as it diminishes the metastatic potential of breast cancer cells [156], but in the case of the overexpression of MMPs 2 and 9, which indicates an unfavourable prognosis as it degrades type 4 collagen, located in the basal membranes, and induces the expression of angiogenic factors [157].

The local invasion of the tumours depends on the degradation of the proteins of the basal membranes, such as type 4 or 5 collagen, and the proteolysis of type 1, 2, or 3 interstitial collagen present in the connective tissue that surrounds the tumour cells [158]. In addition, the MMPs intervene in angiogenesis, promoting the migration of the endothelial cells, liberating VEGF and other proangiogenic factors of the ECM, such as FGF-2 and TGFβ, which also favour the proliferation and migration of these cells [159, 154]. In animal models, it has been demonstrated that the MMPs regulate the formation and maturation of new blood vessels through the control they exercise on the growth factors and cytokines, which act on the recruitment of pericytes [160]. Some MMPs can have an inhibiting effect on angiogenesis; for example, the hydrolysis of plasminogen creates fragments of angiostatin and the proteolysis of XVIII collagen produces endostatin [161]. The transcription of the MMPs is induced by inflammatory cytokines, such as IL-1, IL-6, TNF-α, and growth factors such as EGF, HGF, and TGF-β, giving them a preponderant role in the chronic inflammation which is present in the tumour microenvironment. Other factors, such as TGF-α and IL-4 inhibit their expression, and can be considered as therapeutic targets for cancer [162].

## Angiogenesis

Angiogenesis is a crucial mechanism in the development of cancer due to the growing need for oxygen and nutrients, as the lack of these will lead to the latency and death of the tumour cells [163]. Tumour angiogenesis depends on both the angiogenic growth factors, with stimulants such as angiogenin, angiopoietin-1, cyclo-oxygenase, hepatocyte growth factor, and tumour growth factor produced by the tumour cells and those of the host, and the antiangiogenic factors, which are inhibitors and include angiopoietin-2, angiostatin, interferon-α/β endostatin, and vasostatin, which keep the existing blood vessels in a state of quiescence [164].

The vascularisation of the tumour requires the participation of many types of cells in the tumour microenvironment, including the vascular endothelial cells, the pericytes, and the bone marrow precursor cells, whose participation is regulated by hypoxia [165, 166]. Added to these are the tumour-associated macrophages, the tumour-associated fibroblasts, and the mesenchymal mother cells, which also contribute to the vascularisation of the tumour through the liberation of pro-angiogenic signals in the tumour microenvironment. In patients with advanced, vascularised breast cancer, a high mobilisation of mesenchymal mother cells was found in the circulation, which indicates that these cells have an active participation in tumour progression [167, 168]. Lymphangiogenesis is another route for tumour vascularisation, as the cancer cells can also be disseminated through the lymph vessels [169]. In human cervical cancer, the activated macrophages can produce the VEGF-C and VEGF-D factors, which correlate with lymphangiogenesis [170].

In order to initiate neo-vascularisation, an avascular tumour must acquire an angiogenic phenotype that permits it to ‘turn on’ the angiogenic interruptor [171]. Obviously, the mechanism through which the angiogenic interruptor is ‘connected’ must involve partial oxygen pressure sensors that are sufficiently sensitive to rapidly detect the hypoxia produced in the interior of the solid avascular tumour [172]. The response to hypoxia is the induction of the gene that codes the VEGF protein, considered the most potent angiogenic inducer, while it is also very likely that other factors exist, which can play a role in the response of the tumour to hypoxia [173]. It is accepted that the angiogenic interruptor is ‘turned off’, while the effect of the pro-angiogenic molecules is suspended by the antiangiogenic molecules, and the interruptor turns on when the net balance shifts in favour of angiogenesis. The net angiogenic activity of a tumour is the result of the disequilibrium between stimulating and inhibiting signals [174].

## Tumour latency and progression

Tumours can remain in a latent state for years when there is an equilibrium between proliferation and apoptosis, a phenomenon that can be defined ‘as a state of temporal latency of the arrest of tumour growth’ [175]. This condition can be divided into three categories:

### (a) Cellular latency

Structural components of the niche can promote the survival of the tumour cells while keeping them in a state of latency [176]. Such is the case of the cytoskeleton, which can reactivate these cells, which suggests that the rigidity of the extracellular matrix presents the property of promoting the exit from latency. In an *in vitro* model using cells from a hepatocellular carcinoma, the increase in rigidity of the microenvironment was linked to the TGF-ß factor through induction of the D1 and D3 cyclins [177], showing that less rigid environments favour the latency of tumour cells. In a breast cancer model, it was shown that TGF-ß1 induced fibrosis through deposition of type 1 collagen in the lung, which favoured the escape from latency [178]. In breast cancer, the fibroblasts associated with the cancer can interact with proteins in the extracellular matrix to modulate the intracellular adhesions, cellular contractibility, and forces within the tumour microenvironment. It has been demonstrated that caveolin-1, or Cav-1, produced by the fibroblasts promotes the rigidity of the tumour microenvironment through the activation of GTPasa, thus stimulating tumour progression [179]. The induction of the EMT within the niche causes the tumour cells to enter into a state of latency due to the increase in the levels of p16INK4a [180], the repression of cyclin D [181] and a sustained expression of twist [182]. The latency confers resistance to tumour cells against the action of antitumour agents, whether due to arrest of the cell cycle or a slow proliferation which makes the action of the antitumour agents ineffective [183]. On the other hand, the latent cells must be reactivated in order to grow and form a metastasis. Once the niche has matured, some of its components may act to liberate the cells from latency. Changes in the cytokines can liberate the latent cells, induced by the TCD4^+^ cells [184]. Also, the remodelling of the extracellular matrix by type 1 collagen has been implicated in the liberation from latency through integrin signalling mediated by FAK [185]. The uPAR urokinase receptor activates the β1 integrin, which on interacting with fibronectin, permits the liberation of the tumour cell from latency [186].

### (b) Angiogenic latency

As with normal tissues, the tumours provide nutrients to the cells, such that an inefficient vascularisation causes the tumour mass to remain constant due to an equilibrium between the cells that are dividing and those which are dying. This is the reason why tumour cells, when forming a micrometastasis, must vascularise it in order to survive, as if this does not occur, it can disappear or enter into a state of latency [187], a condition in which it will remain until genetic, epigenetic, and microenvironmental signals can activate angiogenesis. In a study of immunodeficient mice who carried a liposarcoma that was able to remain latent for more than 90 days, high levels of thrombospondin (TSP) and angiomotin were demonstrated [188]. Thrombospondin or TSP is a glycoprotein of the cellular matrix which in physiological conditions is segregated by the fibroblasts and other cells, such as the endothelial cells [189]. On the other hand, TSP was found in latent breast cancer tumour cells that were in contact with the microvasculature of the organs in which they would have metastasised. In a three-dimensional *in vitro* model, the endothelial cells that are part of the vascular system produce the TSP, which acts as a suppressor of angiogenesis and causes the tumour cells to remain in a state of latency. This can explain the effect of TSP on the latency of tumour cells [190]. TSP is a suppressor protein of angiogenesis, however, with the formation of new vessels the endothelial cells produce TGF-ß1 and periostin (POSTN), which will permit the latent cells to initiate their proliferation [191]. The overexpression of Notch-delta 4 (DLL4) in endothelial cells can promote the exit of T-ALL cells from a latent state through joining with the Notch 3 receptor. This overexpression of DLL4 can be induced by the vascular endothelial growth factor VEGF, with the resulting exit from the latent state [192].

### (c) Immunologic latency

A critical step in tumour progression is the evasion and suppression of the immune system of the host [193], which can be achieved through the inhibition of immune effector cells or through the stimulation of immunosuppressor cells. One of the most common mechanisms of immune evasion in patients is due to the activity of the myeloid-derived suppressor cells or MDSCs, which are defined as immature immunosuppressant myeloid cells that maintain the homoeostasis of normal tissue in response to adverse situations such as infections, post-traumatic stress, etc [194]. During tumorigenesis, the MDSC cells infiltrate the tumour promoting its vascularisation [195] and disturbing the immune mechanisms such as the production of antigens by dendritic cells or DCs [196], the activation of T cells [197], the polarisation of M1 macrophages [198] and the inhibition of the cytotoxic NK cells [199]. The MDSC cells promote tumour progression, which has been demonstrated in animal models, and is supported by the elevated number of these cells which is found in patients with cancer, which correlates with an advanced stage of the disease and treatment failure [200].

## Stroma and growth of the tumour cells: pre-clinical studies

As we have seen, in a tumour the different cells of the stroma can interact directly with each other through direct contact or through paracrine signalling. In particular case of the tumour cells, these can induce changes in the cells of the stroma and the extracellular matrix through several mechanisms. They can also alter the stroma through cell–cell contact, modify the extracellular matrix, or liberate soluble factors. They often secrete cytokines, chemocytokines, growth factors, metalloproteinases, and inflammatory mediators that can promote invasivity. However, the mechanisms through which the stroma cells facilitate the growth of tumour cells are not known, and further studies are needed to understand the mechanisms implicated between the cells and the tumour microenvironment in order to design new strategies against cancer and improve existing treatments. Preclinical studies have tested treatments blocking the mechanisms that the tumour cells develop to evade the immune response. Ipilimumab, an antibody that activates the T cells, promoting antitumour activity, was tested on patients with metastatic melanoma, and increased their survival in comparison to patients not treated with this antibody [201]. The CD40 antibody reverses immune suppression activating the antigen-producing cells and promoting the antitumour response of the T cells [202]. An elevated expression of programmed cell death protein 1, PD-1, is produced with cancer.

It has been demonstrated that Pembrolizumab or MK-3475 blocks PD-1 and its ligand PD-L1 expressed in tumour cells in the tumour microenvironment, both in melanomas and in other types of tumours [203]. Dasatinib, an Src gene inhibitor, makes it difficult for melanoma tumour cells to survive [204] as CSF-1R, a regulator or the macrophages associated with tumours, inhibits apoptosis, migration, and invasion in the breast cells of canines [205]. Nintedanib is a simultaneous inhibitor of VEGFR, PDGFR, and FGR, which shows antiangiogenic and antineoplasic inhibitory activity in lung cancer, impeding the growth of tumour cells [206].

In addition, products have been used, which impede the degradation of bone to avoid its colonisation by tumour cells, as is the case with prostate cancer. Micro RNA, miR-34^a^, acts as a suppressor of osteoclastogenesis, bone resorption, and the formation of a metastatic niche, impeding the establishment of metastatic cells [207]. Resveratrol inhibits the EMT transition in the cellular line of LoVo colon cancer through the inhibition of the TGF-β1/Smads pathway mediated by the expression of Snail/E-cadherin, impeding invasion and metastasis [208]. Curcumin inhibits the EMT transition and induces apoptosis in PANC-1 pancreatic cells through the Shh-GLI1 pathway [209].

## Conclusion

Cancer is not only the transformation of individual cells into a state of cellular proliferation, but a disruption of the forms in which the tissues regulate their processes and affect the systemic interactions with the affected organism. Currently, the fundamental treatments against cancer continue to be surgery, radiation therapy, and chemotherapy, which usually destroys the primary tumour, but whose action is very limited against metastasis. This is why it is necessary to continue investigating to find new prognostic markers and new therapeutic targets for metastasis before it occurs, as the early detection of these markers could determine which cases require treatment and avoid it in those patients without a risk of metastasis. Thus, for example, the monitoring of growth factors and cytokines in the blood which may induce the formation of the premetastatic niche would be fundamental. At the same time, determining the blood levels of components of the metastatic niche such as the VEGFR1 protein circulating or interfering with the formation of inflammatory components such as type CD11b^+^ myeloid cells is indispensable for this purpose.

Genetic, cellular biology, and molecular studies, as well as those of the internal and external environmental contexts, indicate that tumour growth is not only determined by its cells, but also by the tumour microenvironment and the entire context in which the organism functions. In this way, the progression of cancer is the result of a very complex relationship between the different malignant and non-malignant cell types, components of the stroma, and the entire body of the organism.

Due to the implication of metastasis in mortality due to cancer, it is also necessary to search for new ways to integrate the two focuses which dominate the current science: the reductionist vision and the systemic vision sustained by the science of complexity.
